# Anniversary Dinner of the Society for the Relief of Widows and Orphans of Medical Men

**Published:** 1848-01

**Authors:** 


*Anniversary Dinner of the Society for the relief of Widows and Orphans of Medical Men.*—This benevolent and prosperous association in the city of New-York, have lately celebrated the anniversary of the formation of the Society, by a dinner at the Astor House. Good feeling, good cheer, and good speeches appear to have united in rendering the occasion all that was to have been expected. Apart from the objects, which commend themselves in the strongest manner to the feelings of every medical man, such occasions are productive of the happiest influences upon the profession. We should be glad to see a custom of meeting for social purposes at stated periods, become more general among medical men than it now is. The better Physicians know each other by means of intimate, friendly companionship, the greater will be their mutual respect for each other, the more highly will they esteem their common profession, and the stronger will be their claims, as a body, upon public regard and confidence. *Fraternal union*—this is the *open sesame*, for secret stores of honor and usefulness, which medicine may possess by never forgetting to avail itself of that talismanic expression.

